# Antibiotic susceptibility of human-associated *Staphylococcus aureus* and its relation to *agr* typing, virulence genes, and biofilm formation

**DOI:** 10.1186/s12879-021-06307-0

**Published:** 2021-07-01

**Authors:** Safoura Derakhshan, Masoumeh Navidinia, Fakhri Haghi

**Affiliations:** 1 Liver and Digestive Research Center, Research Institute for Health Development, Kurdistan University of Medical Sciences, Sanandaj, Iran; 2 School of Allied Medical Sciences, Shahid Beheshti University of Medical Sciences, Tehran, Iran; 3 Department of Microbiology and Immunology, Faculty of Medical Sciences, Zanjan University of Medical Sciences, Zanjan, Iran

**Keywords:** *Staphylococcus aureus*, Drug resistance, Agr protein, Virulence genes, Biofilm

## Abstract

**Background and objective:**

Carriage of virulence factors confers some evolutionary benefit to bacteria, which favors the resistant strains. We aimed to analyze whether antibiotic susceptibility of *Staphylococcus aureus* strains is affected by *agr* typing, biofilm formation ability, and virulence profiles.

**Methods:**

A total of 123 *S. aureus* clinical isolates were subjected to antimicrobial susceptibility testing by disk diffusion method, biofilm formation by microtiter plate method, as well as polymerase chain reaction screening to identify virulence genes and the accessory gene regulator (*agr*) types I-IV. A *P* value < 0.05 was considered significant.

**Results:**

The most prevalent virulence gene was staphyloxanthin *crtN*, followed by hemolysin genes, capsular *cap8H*, toxic shock toxin *tst*, and enterotoxin *sea*, respectively. Resistant isolates were more commonly found in the *agr*-negative group than in the *agr*-positive group. Isolates of *agr* type III were more virulent than *agr* I isolates. Strong biofilm producers showed more antibiotic susceptibility and carried more virulence genes than non-strong biofilm producers. Associations were found between the presence of virulence genes and susceptibility to antibiotics. Carriage of the virulence genes and *agr* was higher in the inpatients; while, resistance and strong biofilms were more prevalent in the outpatients.

**Conclusion:**

These findings indicated the presence of several virulence factors, biofilm production capacity, *agr* types and resistance to antibiotics in clinical *S. aureus* isolates. Considering the importance of *S. aureus* for human medicine, an understanding of virulence and resistance relationships would help to reduce the impact of *S. aureus* infections.

## Introduction

During the past decade, the rise in the emergence and spread of antimicrobial resistance among the microorganisms has become a major concern worldwide. Genetic determinants of resistance are often located on mobile elements and can be easily transmitted between different hosts [1].

Antimicrobial resistance is also a major threat to public health in *Staphylococcus aureus* infections. The first case of methicillin-resistant *S. aureus* (MRSA) was described in the United Kingdom in the 1960s, shortly after the introduction of methicillin into clinical practice [2].

*S. aureus* is also known for its wide range of virulence factors and its ability to infect almost any part of the body. These factors can be divided into different groups, including surface-associated factors, hemolysins and enzymes, and superantigenic toxins [3]. One of the most important surface-associated factors is capsule, which is essential for adherence and initial stages of infection, and can bind to a variety of abiotic surfaces [4]. Some *S. aureus* isolates secrete pyrogenic toxin superantigens, such as toxic shock syndrome toxin 1, exfoliative toxins and enterotoxins. Superantigens are able to activate large numbers of T lymphocytes with a massive production of cytokines and chemokines [5]. Hemolysins are cytotoxins that kill erythrocytes and/or phagocytes by forming pores in cell membranes and also provide nutrients required for growth and spreading of the pathogen through the body. Furthermore, staphyloxanthin, a golden pigment produced by more than 90% of *S. aureus* isolates [6, 7] was suggested to act as an important virulence factor and its inhibition has been shown to reduce the virulence of *S. aureus* [7, 8].

Expression of most virulence factors in *S. aureus* is tightly regulated by the accessory gene regulator (*agr*) locus, which encodes a two-component signaling pathway. *S. aureus* isolates can be divided into four major *agr* groups: types I to IV [9]. The activation of the *agr* system switches the bacterium from being a sessile colonizer to an invasive, aggressive pathogen [10]. However, studies have shown that *agr*-negative strains can persist during infections and may be associated with greater difficulties in management [11].

Human-associated clinical *S. aureus* strains may be naturally deficient in a scale of putative virulence determinants. Reduced toxicity can hide bacteria from the immune system, and facilitate colonization in the host. Therefore, the numbers and combinations of virulence genes may contribute to the pathogenesis of *S. aureus* infections [12].

Most types of virulence factors in *S. aureus* are encoded in mobile genetic elements which can be transferred between bacteria by horizontal gene transfer. Horizontal gene transfer is one of the main mechanisms for the dissemination and co-selection of virulence and resistance which is involved in bacterial evolution and adaptation [5]. The exchange and transfer of genes are also facilitated by biofilm formation. As biofilms are resistant to clearance by immune system or antibiotics, they contribute to the persistent and difficult-to-treat character of staphylococcal diseases [13].

The *agr* system in *S. aureus* is a dominant regulator of pathogenesis and represents a clear example of the link between resistance and virulence. It can control the expression of virulence factors and antimicrobial resistance genes. On the other hand, virulence and antimicrobial resistance genes can up-regulate this system. However, there is an overly complex relationship between virulence and antibiotic resistance. Multiple factors associated with bacteria and their environments affect the evolution of antibiotic resistance and virulence [1, 13].

As monitoring resistance and virulence profiles is important to establish control strategies, we aimed to analyze whether antibiotic susceptibility of *S. aureus* clinical strains is affected by *agr* typing, biofilm formation ability, and virulence profiles.

## Materials and methods

### Bacterial isolates and identification

A total of 123 non-duplicate *S. aureus* isolates were collected from different clinical specimens of patients admitted to 6 teaching hospitals in 3 cities in Iran. The cities were Sanandaj, Zanjan and Tehran (the capitals of 3 different provinces), which were distributed in distant geographic areas in Iran.

Identification of *S. aureus* was performed by standard tests including colony morphology, Gram staining, catalase production, sensitivity to furazolidon, mannitol fermentation, coagulase production and DNase test [14]. All *S. aureus* isolates were further confirmed by polymerase chain reaction (PCR) using primers: nuc1 (5′- GCGATTGATGGTGATACGGTT − 3′) and nuc2 (5′-AGCCAAGCCTTGACGAACTAAAGC − 3′) (amplicon size: 279 bp), to amplify a portion of the thermostable nuclease gene (*nuc*) of *S. aureus* [15]. The PCR program was initial denaturation at 94 °C for 5 min; 35 cycles of denaturation at 94 °C for 30 s, annealing at 61 °C for 30 s, and primer extension at 72 °C for 30s; and a final extension step at 72 °C for 5 min. *S. aureus* ATCC 25923 was used as the control strain.

Dissimilarities between the isolates were assessed using the random amplification of polymorphic DNA (RAPD)-PCR [16]. All isolates were stored at − 70 °C in Trypticase soy broth (TSB, Que-lab, Canada), containing 15% glycerol for further investigation.

### Antimicrobial susceptibility determination

The isolates were assessed for antimicrobial susceptibility by the disk diffusion method according to the 2019 Clinical and Laboratory Standards Institute (CLSI) guidelines [17]. The antimicrobial agents were erythromycin (15 μg), clindamycin (2 μg), gentamicin (10 μg), tetracycline (30 μg), trimethoprim-sulfamethoxazole (1.25–23.75 μg), linezolid (30 μg), and ciprofloxacin (5 μg). MRSA isolates were identified using cefoxitin disk (30 μg) (all from Rosco, Denmark).

Briefly, a bacterial suspension with a turbidity equivalent to the McFarland 0.5 standard (1.5× 10^8^ colony forming unit/mL) was prepared. Antibiotic disks were placed onto the inoculated Mueller-Hinton agar (Que-lab, Canada) plates with the appropriate distances and the plates were then incubated at 35 °C for 16–18 h. The inhibition zones were measured and interpreted according to the 2019 CLSI guidelines [17]. *S. aureus* ATCC 25923 was used as the quality control strain.

### DNA extraction

Genomic DNA was extracted using boiling-freezing method. Briefly, suspension of each isolate in sterile deionized water was boiled for 10 min and immediately placed on ice for 5 min. After three cycles of freezing-thawing, the tubes were centrifuged. The supernatants were removed and stored at − 20 °C as DNA template until experiments. The quantity of each DNA extract was determined by measuring the absorbance at 260 nm to estimate DNA concentration and by calculating the ratio of A260/A280 to determine purity. The DNA was considered to be pure when the A260/A280 ratio was within the range of 1.6–2.0 [18].

### *agr* typing

The *agr* types (I–IV) were determined by PCR using previously described primers [9]. The PCR assay was performed in 25 μL of reaction mixture containing 1X PCR buffer, 1.5 mM MgCl_2_, 200 μM dNTP, 1 unit of Taq polymerase, 0.4 μM of each primer (SinaClon, Iran), and 3 μL of template DNA. The reaction mixtures were subjected to amplification in a thermocycler (Eppendorf, Germany) under the following conditions: an initial denaturation step at 94 °C for 5 min; followed by 35 cycles of denaturation at 94 °C for 1 min, annealing at 55 °C for 1 min, and extension at 72 °C for 1 min; and a final extension step at 72 °C for 5 min. PCR products were analyzed by electrophoresis through 1.5% agarose gel containing DNA safe stain (SinaClon, Iran) and visualized under ultraviolet light.

### Detection of virulence determinants

All isolates were examined by PCR for the presence of 14 genes encoding staphyloxantin (*crtN*), surface factors (capsular types 8 and 5), hemolysins (*hla*, *hlb*, and *hld* genes), and superantigenic toxins [classical enterotoxins (*sea*, *seb*, *sec*, *sed*, and *see*), toxic shock syndrome toxin-1 (*tst*), and exfoliative toxins (*eta* and *etb*)] on a thermocycler (Eppendorf, Germany).

The PCR reactions were performed in a final volume of 25 μL consisted of 1 μg of genomic DNA, 0.4 μM of each primer, and 12.5 μL of 2X Taq PCR Master Mix (SinaClon, Iran: 0.08 U of Taq polymerase/ μL, 0.4 mM of each dNTP, and 3 mM MgCl_2_). The cycling conditions were as follows: an initial denaturation at 94 °C for 5 min; 35 cycles of denaturation at 94 °C for 1 min, annealing at different temperatures for 1 min (Table 1), and primer extension at 72 °C for 1 min; and a final extension at 72 °C for 5 min. The PCR products were analyzed by electrophoresis on a 1.5% agarose gel. The target genes, primer sequences, their respective amplified products and annealing temperatures are shown in Table 1.

**Table 1:** Genes, primer sequences, annealing temperatures and predicted size of PCR products

Gene	Protein	Primer sequence (5′-3′)	Product size (bp)	^**1**^Annea.(°C)	^**2**^Ref.
*crtN*	Staphyloxanthin	ATTGATCCAAAACGAGGCCC/ CGGCCCGTTTGAATTTAGGA	196	55	This study
**Surface factors**					
*cap5H*	CP5 synthesis enzyme	ATGAGGATAGCGATTGAAAA/ CGCTTCTTAATCACTTTTGC	518	50	[3]
*cap8H*	CP8 synthesis enzyme	ATCGAAGAACATATCCAAGG/ TTCATCACCAATACCTTTTA	834	49	[3]
**Hemolysins**					
*hla*	Alfa toxin	TGCCGCAGATTCTGATATTAA/ TTTCTGAAGAACGATCTGTCCA	845	55	[3]
*hlb*	Beta toxin	GCGGTTGTGGATTCGATAAT/ GGCTTTGATTGGGTAATGATC	524	55	[3]
*hld*	Delta toxin	GGGATGGCTTAATAACTCATACTT/ CAGAGATGTGATGGAAAATAGTTGA	236	62	[3]
**Superantigenic toxins**					
*tst*	Toxic shock syndrome toxin-1	ACCCCTGTTCCCTTATCATC/ TTTTCAGTATTTGTAACGCC	326	52	[19]
*eta*	Exfoliative toxin A	CTAGTGCATTTGTTATTCAA/ TGCATTGACACCATAGTACT	119	50	[20]
*etb*	Exfoliative toxin B	ACAAGCAAAAGAATACAGCG/ GTTTTTGGCTGCTTCTCTTG	226	52	[19]
*sea*	Enterotoxin A	GGTTATCAATGTGCGGGTGG/ CGGCACTTTTTTCTCTTCGG	102	56	[19]
*seb*	Enterotoxin B	GTATGGTGGTGTAACTGAGC/ CCAAATAGTGACGAGTTAGG	164	55	[19]
*sec*	Enterotoxin C	AGATGAAGTAGTTGATGTGTATGG/ CACACTTTTAGAATCAACCG	451	52	[19]
*sed*	Enterotoxin D	CCAATAATAGGAGAAAATAAAAG/ ATTGGTATTTTTTTTCGTTC	278	48	[19]
*see*	Enterotoxin E	AGGTTTTTTCACAGGTCATCC/ CTTTTTTTTCTTCGGTCAATC	209	52	[19]

^1^: Annealing temperature, ^2^: reference

### Biofilm formation

Biofilm formation assay was performed as previously described [21]. Overnight cultures of isolates were adjusted to the turbidity of a 0.5 McFarland standard in TSB medium and 100 μL of each dilution was loaded into the wells of a non-treated flat-bottom 96-well microtiter plate (Jet Biofil, China). *S. aureus* ATCC25923 (biofilm forming) and *Staphylococcus epidermidis* ATCC12228 (not biofilm-forming) were used as controls. After 24 h incubation at 37 °C for biofilm production, the supernatants were removed and the wells were washed with distilled water. The adherent cells were stained with an aqueous solution of crystal violet (0.1%, w/v) at room temperature and washed with distilled water. Then, the microtiter plates were dried for a few hours. Bound crystal violet was dissolved by treatment with 30% acetic acid at room temperature and optical density (OD) of each well was measured by using a microplate reader (Anthos Labtec, Netherlands) at 550 nm. The negative control wells contained broth only.

Biofilm density was classified according to the scheme of Stepanovic et al. [22]. The cut-off value (ODc) for each microtiter plate was defined as three standard deviations above the mean OD of the negative control. Isolates were then classified into four categories based on ODc and average OD of the strain: strong biofilm producer (4ODc ≤ OD); moderate biofilm producer (2ODc ≤ OD ≤ 4ODc); weak biofilm producer (ODc ≤ OD ≤ 2ODc), and no biofilm producer (OD ≤ ODc). For biofilm formation assay, 4 wells per strain were used; and each test was repeated three times.

### Statistical analysis

Statistical analyses were performed with the chi-square or Fisher’s test (if necessary) using the SPSS software 16 (SPSS Inc., United States). The significance level was set at 0.05. MRSA isolates or isolates exhibiting non-susceptibility to at least one agent in three or more different antimicrobial categories were defined as multidrug-resistant (MDR) [23]. The isolates were classified as strong biofilm producers or non-strong (moderate and weak) biofilm producers for statistical purposes [24].

## Results

A total of 123 strains were isolated from different clinical specimens. Of these, 68 were isolated from males and 55 were from females. The majority of strains were isolated from urine (61/123, 49.6%), followed by blood (34/123, 27.6%), wound (21/123, 17.1%), and others (7/123, 5.7%). A total of 97 strains were isolated from inpatients, and thus 26 were from outpatients.

### Antimicrobial susceptibility

The most effective antibiotics were linezolid (123/123, 100% sensitivity), trimethoprim-sulfamethoxazole (120 /123, 97.6%), and gentamicin (116/123, 94.3%). The susceptibility rate to other antibiotics was clindamycin 97 (78.9%), ciprofloxacin 96 (78.1%), tetracycline 89 (72.4%), and erythromycin 64 (52%). Fifteen isolates (12.2%) were classified as MRSA by cefoxitin disk diffusion test; thus 108 (87.8%) isolates were methicillin- sensitive *S. aureus* (MSSA). Out of the 123 isolates, 52 (42.3%) were susceptible to all of the antibiotics tested, while 30 isolates (24.4%) were MDR. Resistance patterns ranged from resistance to one antibiotic (*n* = 34) to all antibiotics (*n* = 3). Thirty four isolates (27.6%) displayed resistance to one agent, 10 isolates (8.1%) to two agents, 7 isolates (5.7%) to three agents, 12 isolates (9.8%) to four agents, and 8 isolates (6.5%) showed resistance to five and more agents.

### Detection of virulence genes and biofilm formation

Out of the 123 isolates, 117 isolates (95.1%) carried at least one of the 14 tested virulence genes. The most prevalent virulence gene was *crtN* (110/123, 89.4%), followed by hemolysin genes (*hld*: *n* = 93, 75.6%; *hlb*: *n* = 83, 67.5%; and *hla*: *n* = 48, 39%), *cap8H* (*n* = 29, 23.6%), *tst* (*n* = 28, 22.8%), and *sea* (*n* = 17, 13.8%), respectively. The least commonly detected virulence genes were *see* (*n* = 5, 4.1%), *cap5H* (*n* = 3, 2.4%), *eta* (*n* = 2, 1.6%), and *seb* (*n* = 1, 0.8%). Additionally the *etb*, *sec*, or *sed* were not found. Therefore, they didn’t appear in statistical analysis of differences among groups. Six isolates (4.9%) carried no virulence gene and a high percentage of strains (91/123, 74%) expressed neither CP5 nor CP8. Thirty different virulotypes were found in the isolates. Number of virulence genes ranged from 1 (*n* = 10 isolates) to 7 genes (*n* = 2 isolates). Carriage of two virulence genes was the most detected virulotype (26/123, 21.1%).

Regarding biofilm formation, all the 123 isolates formed biofilm, of which 27 formed strong biofilms and 96 formed non-strong biofilms (27 isolates formed weak biofilms and 69 formed moderate biofilms).

### *agr* genotyping

Of the 123 isolates, the *agr* types were identified in 80 isolates (65%), while 43 isolates were negative for *agr* locus (35%). The *agr* III was found in 55 isolates (44.7%) and *agr* I in 25 isolates (20.3%). The *agr* types II and IV were not found.

#### Statistical analysis of differences among groups

In case of virulence genes, *eta*, *etb, cap5H*, *seb*, *see*, *sec*, and *sed* and in case of antibiotics, linezolid, trimethoprim-sulfamethoxazole, and gentamicin were excluded from statistical analysis of differences among groups.

### Virulence genes and antibiotic susceptibility phenotype in *agr* groups

**Fig. 1:**
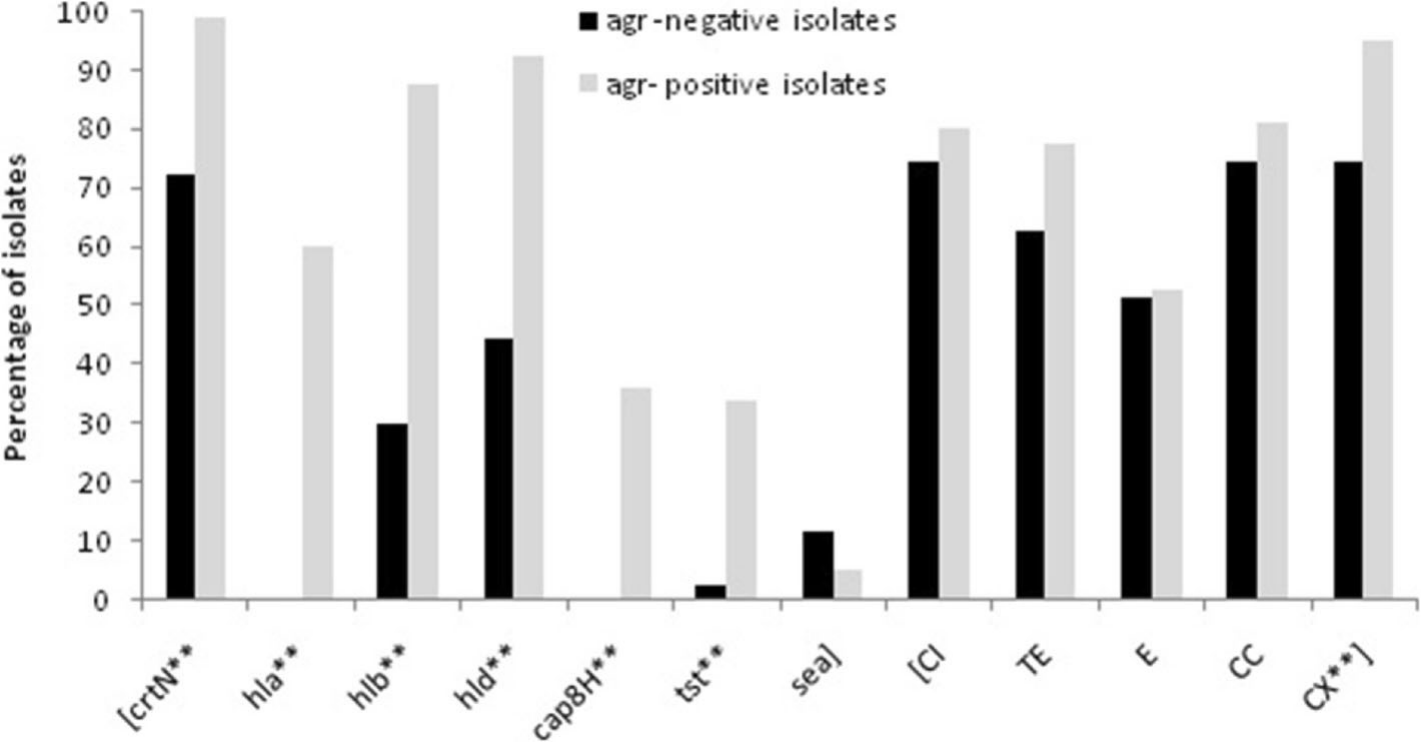
Distribution of virulence- associated genes and antibiotic susceptibility phenotype between *agr*-positive (*n* = 80) and *agr*-negative (*n* = 43) *S. aureus* clinical isolates. X^2^ test and Fisher’s test were used for comparisons. **: *P* ≤ 0.001. CI, ciprofloxacin; TE, tetracycline; E, erythromycin; CC, clindamycin; CX, cefoxitin

Figure 1 shows the distribution of virulence genes and susceptibility to antibiotics, according to the *agr* typing of isolates. The *agr*-positive isolates showed a significantly higher prevalence of the virulence genes (98.8 to 33.8% of isolates) compared to the *agr*-negative isolates (72.1 to 0%) (*P* ≤ 0.001); but for the *sea*, the *agr*-negative isolates showed a higher prevalence; although it was not significant (*P* > 0.05). Notably, the *agr*-negative isolates were quite negative for the *cap8H* and *hla* genes.

The frequency of susceptibility to antibiotics was also examined. Overall, the *agr*-positive isolates were more susceptible to antibiotics compared to the *agr*-negative isolates and a significant association was found between the presence of *agr* and susceptibility to cefoxitin (95% vs. 74.4%, respectively; *P* ≤ 0.001). MRSA isolates were more commonly found in the *agr-*negative group (Fig. 1).

**Fig. 2:**
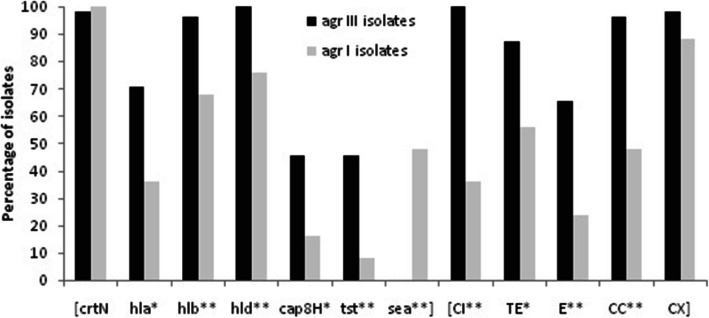
Distribution of virulence genes and antibiotic susceptibility phenotype in *agr* specific groups. X^2^ test and Fisher’s test were used for comparisons. **: *P* ≤ 0.001, *: *P* < 0.05. CI, ciprofloxacin; TE, tetracycline; E, erythromycin; CC, clindamycin; CX, cefoxitin

When comparing virulence genes with *agr* specific groups, significant associations were noted for all genes except *crtN* (Fig. 2). Isolates of *agr* type III more commonly carried *tst*, *cap8H* and hemolysin genes (45.5–100% of isolates) compared to those of *agr* I (8–76%). The *crtN* was almost equally distributed between the two groups; although it was slightly shifted toward *agr* I group and away from *agr* III group. Notably, in contrast to the other genes, *sea* was only found in the *agr* I group.

Furthermore, the possible statistical association between antibiotic susceptibility phenotype and *agr* genotypes was investigated. The *agr* III isolates showed more susceptibility to each agent than the *agr* I group and significant associations were found for each agent except cefoxitin. The susceptibility rate of *agr* III isolates to the tested antibiotics was from 100% to ciprofloxacin to 65.5% to erythromycin. While in the *agr* I group, the most susceptibility was found to cefoxitin (88%) and least to erythromycin (24%).

### Virulence genes and antibiotic susceptibility phenotype in biofilm- producing *S. aureus*

A comparison between the strong and non-strong (moderate and weak) biofilm producers showed that the strong biofilm producers more commonly carried *crtN*, *cap8H* and hemolysin (*hld*, *hlb*, *hla*) genes compared to non-strong biofilm producers (92.6–25.9% of isolates vs. 88.5–22.9%) (*P* > 0.05). But for the *tst* and *sea*, the non-strong biofilm producers carried a higher prevalence and a significant association was found for *tst* (3.7% vs. 28.1%, *P* = 0.008).

To determine whether biofilm formation was correlated with susceptibility to any mentioned antibiotic(s), we compared the biofilm forming capacities among isolates. Although no significant difference was found, susceptibility to the antibiotics was higher in the strong biofilm producers than in the non-strong producers. The highest susceptibility in both groups was seen to cefoxitin (92.6% in the strong group and 86.5% in the non-strong group). The susceptibility rate to the other antibiotics was from 88.9% to clindamycin to 59.3% to erythromycin in the strong biofilm producer group and from 77.1% to ciprofloxacin to 50% to erythromycin in the non- strong biofilm producers.

### Relationship of virulence genes with susceptibility phenotype

Table 2 shows the prevalence of each virulence gene according to the antibiotic susceptibility status of isolates.

**Table 2:** Prevalence of virulence genes (%) according to antibiotic susceptibility in 123 *S. aureus* isolates

^1^Antibiotic	CI		CX		TE		E		CC	
	^**2**^Res. (***n*** = 27)	^**3**^Susc. (***n*** = 96)	Res. (***n*** = 15)	Susc. (***n*** = 108)	Res. (***n*** = 34)	Susc. (***n*** = 89)	Res. (***n*** = 59)	Susc. (***n*** = 64)	Res. (***n*** = 26)	Susc. (***n*** = 97)
Virulence genes										
*crtN*	81.5	91.7	60	**93.5	85.3	91	84.7	93.8	84.6	90.7
*hla*	22.2	*43.8	6.7	*43.5	26.5	43.8	33.9	43.8	23.1	43.3
*hlb*	44.4	*74	46.7	70.4	64.7	68.5	57.6	*76.6	50	*72.2
*hld*	63	79.2	53.3	*78.7	61.8	*80.9	74.6	76.6	61.5	79.4
*cap8H*	11.1	27.1	0	*26.9	17.6	25.8	22	25	15.4	25.8
*tst*	7.4	*27.1	6.7	25	11.8	27	16.9	28.1	11.5	25.8
*sea*	51.9	**3.1	20	13	32.4	**6.7	25.4	**3.1	53.8	**3.1

^1^: CI, ciprofloxacin; CX, cefoxitin; TE, tetracycline; E, erythromycin; CC, clindamycin. ^2^: Resistant isolates; ^3^: Susceptible isolates. X^2^ test and Fisher’s exact test were used for comparisons. **: *P* ≤ 0.001, *: *P* < 0.05

Except for the *sea*, the susceptible isolates showed a higher prevalence of the virulence genes than the resistant isolates (Table 2). Significant associations were found between the presence of *sea* and resistance to all antibiotics (*P* ≤ 0.001), but it was not significant for cefoxitin (*P* > 0.05). The *sea* was found in 20% of MRSA isolates and in 13% of MSSA isolates. For the other virulence genes, the MSSA isolates showed more virulence compared to the MRSA isolates and significant associations were found between susceptibility to cefoxitin and presence of *hla*, *hld*, *crtN* and *cap8H* (*P* < 0.05). Notably, the *cap8H* was only found in MSSA group. In addition, associations were found between the presence of *hla*, *hlb*, and *tst* and susceptibility to ciprofloxacin, *hld* and susceptibility to tetracycline, and *hlb* and susceptibility to erythromycin and clindamycin (*P* < 0.05).

### Virulence genes, antibiotic susceptibility, biofilm formation and *agr* presence in outpatients and inpatients

**Fig. 3:**
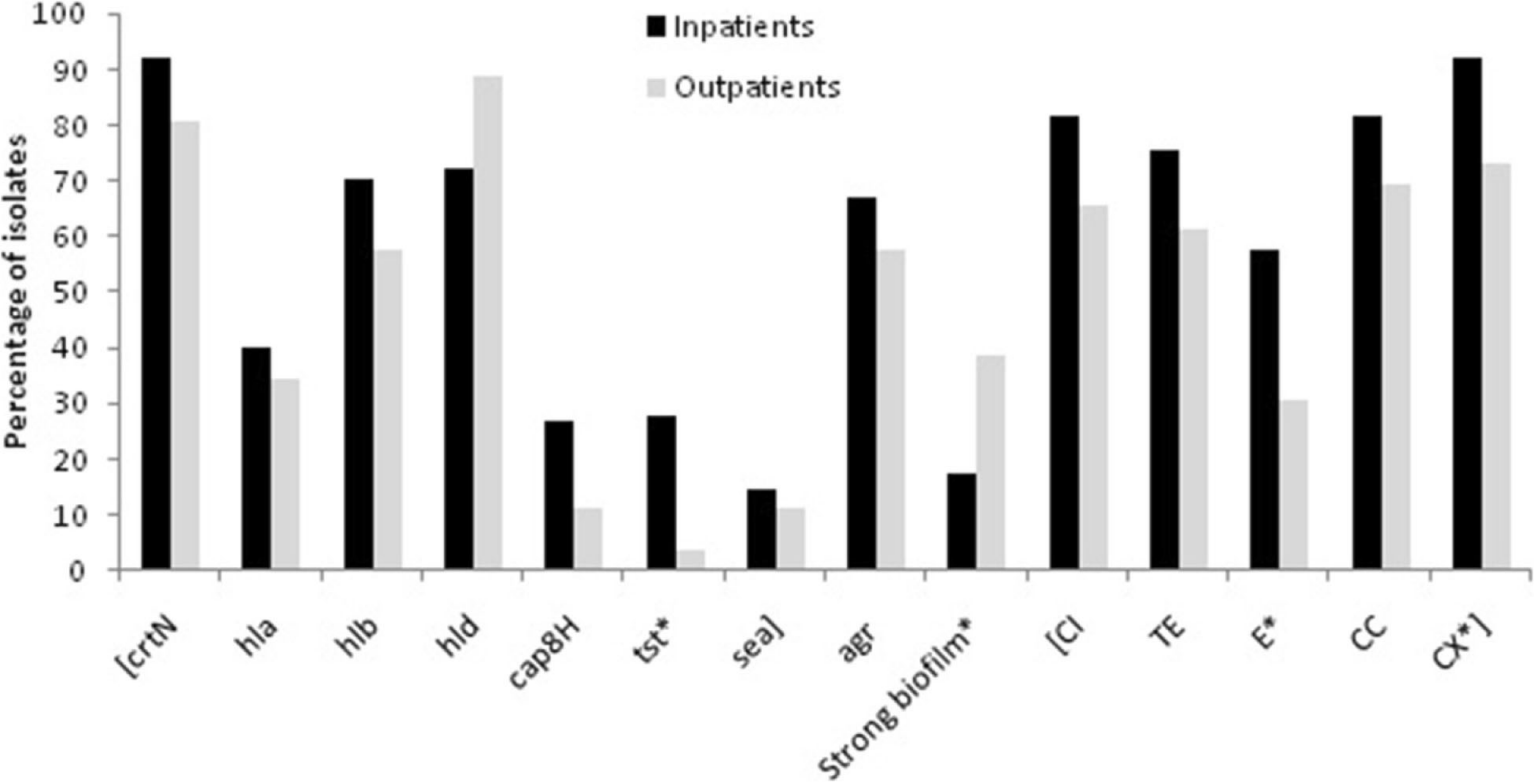
Distribution of virulence genes, antibiotic susceptibility, biofilm formation and *agr* between inpatients and outpatients. X^2^ test and Fisher’s test were used for comparisons. *: *P* < 0.05. CI, ciprofloxacin; TE, tetracycline; E, erythromycin; CC, clindamycin; CX, cefoxitin

As shown in Fig. 3, except for the *hld*, the prevalence of *agr* and virulence genes was higher in the inpatients than in the outpatients and a significant association was found for *tst* (27.8% vs. 3.8%; *P* = 0.01). Notably, prevalence of strong biofilms was significantly higher in the outpatients, while isolates from the inpatients more commonly produced non-strong biofilms.

The frequency of susceptibility to antibiotics in each group was also examined. Interestingly, isolates from the inpatients were more susceptible to antibiotics than those from the outpatients. While there was a significant association between the inpatient status and susceptibility to erythromycin and cefoxitin (*P* = 0.015 and *P* = 0.017, respectively), no statistical significance was found for the other antibiotics (Fig. 3).

## Discussion

*S. aureus* is a major human pathogen that causes a diverse range of hospital and community-acquired infections [8]. The elderly, infants, and immunocompromised patients are at higher risk of *S. aureus* infections [25].

Overall, our results revealed high antibiotic susceptibility rates, which were higher than those reported in two different regions of Iran [12], in China [26], and in Nigeria [27]. Similarly, the prevalence of MRSA and MDR in our study was lower than in the above studies. These variations in the prevalence rates may be due in part to the different regional antimicrobials prescription, infection prevention and control activities, and source of isolates [28].

*S. aureus* produces many virulence factors, located on the plasmid, phage or chromosomes [3, 5]. The predominant CP types in *S. aureus* were reported to be the 5 and 8, which are expressed by about 70% of clinical isolates [3]. We observed a higher prevalence of *cap8H* compared to the *cap5H*. A high percentage of our isolates (74%) expressed neither CP5 nor CP8 which is in contrast to a previous finding [10]. Loss of CP5 or CP8 expression may enhance the persistence of staphylococci in the infected hosts [29]. In accordance with other studies [6, 7], we found a high prevalence of the staphyloxanthin *crtN* gene (89.4%), which has been associated with human clinical diseases [7, 8]. The superantigenic toxins and hemolysins play important roles in modulation of the host immune response [5]. Different prevalence rates of 0 to 80% were reported for these toxins in different regions [8, 10, 12, 26, 30]. Therefore, a considerable variation may be observed in the prevalence of these toxins due to the geographical location or source of isolates.

In *S. aureus*, the *agr* locus is a dominant regulator of virulence and pathogenesis [9]. We found that all of the tested virulence genes except *sea* were associated with *agr*-positive strains. Consistent with our finding, presence of the *agr* operon was strongly associated with the carriage of virulence genes and a higher survival rate of nematodes exposed to *agr*-negative strains was found compared to those exposed to *agr*-positive strains, highlighting the difference in pathogenicity between *agr*-positive and *-*negative strains [10]. It has been noted, however, that *agr*-negative *S. aureus*, although not capable of causing severe infections, would initiate and establish colonization. There are studies that have linked a loss of *agr* functionality to diminished glycopeptides activity and prolonged infections [11, 31]. Our *agr*-negative isolates showed more resistance to antibiotics especially to cefoxitin. These features may be favorable to *agr* negative strains, although the virulence repertoires of such strains were lower compared to the *agr*-positive strains. There are discrepancies among the different studies about the role of *agr* groups in antibiotic treatment. Our *agr* III isolates showed more antibiotic susceptibility than *agr* I isolates. A study in Pakistan showed that *agr* III isolates were more resistant than *agr* I isolates [32]; whereas, in Iran, a significant relation was found between *agr* I and resistance to cefoxitin and erythromycin (*P* < 0.05) [33]. In addition, in our study, *agr* III isolates more commonly carried the mentioned virulence genes compared to *agr* I isolates; but the *sea* was only found in the *agr* I isolates (Fig. 2). Some studies reported that TSST1-producing isolates belonged to *agr* III or exfoliative syndrome was associated with *agr* IV strains [34]; whereas He et al., reported that isolates of *agr* II and IV more commonly carried *tst* than those of *agr* I and III [30].

Except for the *tst* and *sea*, a comparison between the strong and non-strong biofilm producers showed a higher number of virulence genes in the strong biofilm producers. The ability of *S. aureus* to form strong biofilms along with the presence of virulence genes may cause high rates of failure in therapy. Furthermore, although no significant difference was seen, resistance to the antibiotics was higher in the non-strong biofilm producers. This was in agreement with the study of Zhang et al. [4]; who found the high prevalence of resistance to ciprofloxacin, gentamicin, tetracycline, clindamycin, and trimethoprim-sulfamethoxazole in moderate and weak biofilm producers, but in contrast to other studies showing the high resistance in strong biofilm producing *S. aureus* [35, 36]. These results may indicate the importance of drug resistance and some virulence genes such as *tst* and *sea* in the pathogenesis of non-strong biofilm producers.

Previously reported data suggest that methicillin resistance in *S. aureus* influences the biofilm phenotype and attenuates virulence [37]. We found an association between susceptibility to antibiotics and presence of virulence genes; but for the *sea*, the resistant isolates showed a higher prevalence (Table 2). These findings indicated that the less virulent genotypes may be predictors of primary antibiotic resistance in patients. It would be worthwhile to investigate whether gene linkage on plasmids or other mobile genetic elements underlies the associations observed for the *sea*. The relationship between resistance and virulence among bacteria depends on the interactions between the multiple factors associated with bacteria and their environments [13]. For instance, Bisognano et al. suggested that use of ciprofloxacin not only selected resistant *S. aureus* strains but also induced the production of virulence factors [38]; while, a relation was found between the reduced susceptibility to vancomycin and the decreased virulence in *S. aureus* [39]. Moreover, it has been noted that virulence repertoire of MRSA strains is different from MSSA strains [28]. whereas, a study on clinical isolates demonstrated no difference in the frequency of virulence genes between MRSA and MSSA isolates [40].

The selection pressure from the concentration of antibiotics in the environment may accelerate the evolution and dissemination of antibiotic-resistant pathogens. However, during infection, the most virulent pathogens will be the most likely to survive [13]. Overall, carriage of the virulence genes and *agr* in our study was higher in the inpatients; while, antibiotic resistance and strong biofilms were more prevalent in the outpatients. Improper and ineffective antibiotic usage may generate a favorable environment for acquisition of more resistant strains, which may explain the higher rate of resistance in the isolates from outpatients [25].

## Conclusion

In summary, these results revealed the presence of several virulence factors, biofilm production capacity, *agr* types and resistance to antibiotics in *S. aureus* isolated from clinical samples. Obviously, microorganisms will be increasingly more resistant to antibiotics in the future. Carriage of virulence genes also confers some evolutionary benefit to bacteria, which thus favors the resistant strains. Considering the high prevalence of infections caused by *S. aureus* and its importance to human medicine, an understanding of virulence and resistance relationships would help to reduce the impact of *S. aureus* infections. Further studies are needed for more detailed information about the different mechanisms of virulence and resistance used by this pathogen and the interaction between them.

## Data Availability

All data generated or analyzed during this study are included in this article.

## Abbreviations

*agr*: accessory gene regulator
MRSA: methicillin-resistant *S. aureus*
MSSA: methicillin- sensitive *S. aureus*
CLSI: Clinical and Laboratory Standards Institute
PCR: Polymerase chain reaction
TSB: Trypticase soy broth
MDR: Multidrug-resistant
bp: Base pairs

## References

[1] Pérez VKC, da Costa GM, Guimarães AS, Heinemann MB, Lage AP, Dorneles EMS. Relationship between virulence factors and antimicrobial resistance in *Staphylococcus aureus* from bovine mastitis. J Glob Antimicrob Resist. 2020;22:792–802. 10.1016/j.jgar.2020.06.010 32603906

[2] Jevons MP (1961). “Celbenin”-resistant staphylococci. Br Med J.

[3] Ote I, Taminiau B, Duprez J-N, Dizier I, Mainil JG (2011). Genotypic characterization by polymerase chain reaction of Staphylococcus aureus isolates associated with bovine mastitis. Vet Microbiol.

[4] Zhang Y, Xu D, Shi L, Cai R, Li C, Yan H (2018). Association between agr type, virulence factors, biofilm formation and antibiotic resistance of Staphylococcus aureus isolates from pork production. Front Microbiol.

[5] Fueyo J, Mendoza MC, Rodicio MR, Muniz J, Alvarez M, Martín M (2005). Cytotoxin and pyrogenic toxin superantigen gene profiles of Staphylococcus aureus associated with subclinical mastitis in dairy cows and relationships with macrorestriction genomic profiles. J Clin Microbiol.

[6] Mishra NN, Liu GY, Yeaman MR, Nast CC, Proctor RA, McKinnell J, Bayer AS (2011). Carotenoid-related alteration of cell membrane fluidity impacts Staphylococcus aureus susceptibility to host defense peptides. Antimicrob Agents Chemother.

[7] Xue L, Chen YY, Yan Z, Lu W, Wan D, Zhu H (2019). Staphyloxanthin: a potential target for antivirulence therapy. Infect Drug Resist.

[8] Zhang J, Suo Y, Zhang D, Jin F, Zhao H, Shi C (2018). Genetic and virulent difference between pigmented and non-pigmented Staphylococcus aureus. Front Microbiol.

[9] Shopsin B, Mathema B, Alcabes P, Said-Salim B, Lina G, Matsuka A, Martinez J, Kreiswirth BN (2003). Prevalence of agr specificity groups among Staphylococcus aureus strains colonizing children and their guardians. J Clin Microbiol.

[10] Thompson TA, Brown PD (2017). Association between the agr locus and the presence of virulence genes and pathogenesis in Staphylococcus aureus using a Caenorhabditis elegans model. Int J Infect Dis.

[11] Fowler VG, Sakoulas G, McIntyre LM, Meka VG, Arbeit RD, Cabell CH (2004). Persistent bacteremia due to methicillin-resistant Staphylococcus aureus infection is associated with agr dysfunction and low-level in vitro resistance to thrombin-induced platelet microbicidal protein. J Infect Dis.

[12] Tahmasebi H, Dehbashi S, Arabestani MR (2019). Association between the accessory gene regulator (agr) locus and the presence of superantigen genes in clinical isolates of methicillin-resistant Staphylococcus aureus. BMC Res Notes.

[13] Beceiro A, Tomás M, Bou G (2013). Antimicrobial resistance and virulence: a successful or deleterious association in the bacterial world?. Clin Microbiol Rev.

[14] Tille P. Bailey & Scott's diagnostic microbiology: Elsevier health sciences; 2015.

[15] Zhang K, Sparling J, Chow BL, Elsayed S, Hussain Z, Church DL, Gregson DB, Louie T, Conly JM (2004). New quadriplex PCR assay for detection of methicillin and mupirocin resistance and simultaneous discrimination of Staphylococcus aureus from coagulase-negative staphylococci. J Clin Microbiol.

[16] Kurlenda J, Grinholc M, Jasek K, Wegrzyn G (2007). RAPD typing of methicillin-resistant Staphylococcus aureus: a 7-year experience in a polish hospital. Med Sci Monit.

[17] CLSIC L. Performance standards for antimicrobial susceptibility testing. Clinical and Laboratory Standards Institute. CLSI Supplement M100. 2019.

[18] Khare P, Raj V, Chandra S, Agarwal S (2014). Quantitative and qualitative assessment of DNA extracted from saliva for its use in forensic identification. J Forensic Dent Sci.

[19] Mehrotra M, Wang G, Johnson WM (2000). Multiplex PCR for detection of genes forStaphylococcus aureus enterotoxins, exfoliative toxins, toxic shock syndrome toxin 1, and methicillin resistance. J Clin Microbiol.

[20] Johnson W, Tyler S, Ewan E, Ashton F, Pollard D, Rozee K (1991). Detection of genes for enterotoxins, exfoliative toxins, and toxic shock syndrome toxin 1 in Staphylococcus aureus by the polymerase chain reaction. J Clin Microbiol.

[21] O'Toole GA. Microtiter dish biofilm formation assay. JoVE. 2011;(47):e2437. 10.3791/2437 PMC3182663 21307833

[22] Stepanović S, Vuković D, Hola V, BONAVENTURA GD, Djukić S, Ćirković I (2007). Quantification of biofilm in microtiter plates: overview of testing conditions and practical recommendations for assessment of biofilm production by staphylococci. Apmis..

[23] Magiorakos A-P, Srinivasan A, Carey R, Carmeli Y, Falagas M, Giske C (2012). Multidrug-resistant, extensively drug-resistant and pandrug-resistant bacteria: an international expert proposal for interim standard definitions for acquired resistance. Clin Microbiol Infect.

[24] Horna G, Quezada K, Ramos S, Mosqueda N, Rubio M, Guerra H, Ruiz J (2019). Specific type IV pili groups in clinical isolates of Pseudomonas aeruginosa. Int Microbiol.

[25] Delorme T, Dang D, Garcia A, Nasr P (2019). Genotypic and phenotypic variations in methicillin-resistant Staphylococcus aureus isolates from outpatient, inpatient and nursing homes. J Med Microbiol.

[26] Liang Y, Tu C, Tan C, El-Sayed MAE-G (2019). Antimicrobial resistance, virulence genes profiling and molecular relatedness of methicillin-resistant Staphylococcus aureus strains isolated from hospitalized patients in Guangdong Province, China. Infect Drug Resist.

[27] Alli OAT, Ogbolu DO, Shittu AO, Okorie AN, Akinola JO, Daniel JB (2015). Association of virulence genes with mecA gene in Staphylococcus aureus isolates from tertiary hospitals in Nigeria. Indian J Pathol Microbiol.

[28] Čižman M (2003). The use and resistance to antibiotics in the community. Int J Antimicrob Agents.

[29] Tuchscherr LP, Buzzola FR, Alvarez LP, Caccuri RL, Lee JC, Sordelli DO (2005). Capsule-negative Staphylococcus aureus induces chronic experimental mastitis in mice. Infect Immun.

[30] He W, Chen H, Zhao C, Zhang F, Li H, Wang Q, Wang X, Wang H (2013). Population structure and characterisation of Staphylococcus aureus from bacteraemia at multiple hospitals in China: association between antimicrobial resistance, toxin genes and genotypes. Int J Antimicrob Agents.

[31] Sakoulas G, Eliopoulos GM, Fowler VG, Moellering RC, Novick RP, Lucindo N (2005). Reduced susceptibility of Staphylococcus aureus to vancomycin and platelet microbicidal protein correlates with defective autolysis and loss of accessory gene regulator (agr) function. Antimicrob Agents Chemother.

[32] Khan S, Rasheed F, Zahra R (2014). Genetic polymorphism of agr locus and antibiotic resistance of Staphylococcus aureus at two hospitals in Pakistan. Pakistan journal of medical sciences.

[33] Javdan S, Narimani T, Abadi MSS, Gholipour A (2019). Agr typing of Staphylococcus aureus species isolated from clinical samples in training hospitals of Isfahan and Shahrekord. BMC Res Notes.

[34] Jarraud S, Lyon G, Figueiredo A, Gérard L, Vandenesch F, Etienne J (2000). Exfoliatin-producing strains define a fourthagr specificity group in Staphylococcus aureus. J Bacteriol.

[35] Lin Q, Sun H, Yao K, Cai J, Ren Y, Chi Y (2019). The prevalence, antibiotic resistance and biofilm formation of Staphylococcus aureus in bulk ready-to-eat foods. Biomolecules..

[36] Sun X, Lin Z-W, Hu X-X, Yao W-M, Bai B, Wang H-Y (2018). Biofilm formation in erythromycin-resistant Staphylococcus aureus and the relationship with antimicrobial susceptibility and molecular characteristics. Microb Pathog.

[37] Rudkin JK, Edwards AM, Bowden MG, Brown EL, Pozzi C, Waters EM, Chan WC, Williams P, O’Gara JP, Massey RC (2012). Methicillin resistance reduces the virulence of healthcare-associated methicillin-resistant Staphylococcus aureus by interfering with the agr quorum sensing system. J Infect Dis.

[38] Bisognano C, Kelley WL, Estoppey T, Francois P, Schrenzel J, Li D, Lew DP, Hooper DC, Cheung AL, Vaudaux P (2004). A RecA-LexA-dependent pathway mediates ciprofloxacin-induced fibronectin binding in Staphylococcus aureus. J Biol Chem.

[39] Peleg AY, Monga D, Pillai S, Mylonakis E, Moellering RC, Eliopoulos GM (2009). Reduced susceptibility to vancomycin influences pathogenicity in Staphylococcus aureus infection. J Infect Dis.

[40] Luo K, Shao F, Kamara KN, Chen S, Zhang R, Duan G, Yang H (2018). Molecular characteristics of antimicrobial resistance and virulence determinants of Staphylococcus aureus isolates derived from clinical infection and food. J Clin Lab Anal.

